# Genome-wide high-throughput transposon mutagenesis unveils key factors for acidic pH adaptation of Corynebacterium diphtheriae

**DOI:** 10.1099/mic.0.001554

**Published:** 2025-04-24

**Authors:** Camila Azevedo Antunes, Emily C. A. Goodall, Ian R. Henderson, David Wild, Alexander Mehltretter, Philipp Ott, Markus Hölzl, Lisa Ott, Gerald Seidel, Andreas Burkovski

**Affiliations:** 1 Microbiology Division, Department of Biology, Friedrich-Alexander-Universität Erlangen-Nürnberg, Staudtstr. 5, Erlangen, Germany; 2 Institute for Molecular Bioscience, University of Queensland, Brisbane, Australia

**Keywords:** *Corynebacterium diphtheriae*, *Corynebacterium glutamicum*, diphtheria, host–pathogen interaction, *Mycobacterium*, potassium transport, stress response, TraDIS

## Abstract

*Corynebacterium diphtheriae,* a notable pathogen responsible for the life-threatening disease diphtheria, encounters harsh intracellular environments within the host, particularly within macrophages where acidic conditions prevail. To elucidate the genetic and molecular mechanisms underlying its acid stress response, we employed a Transposon Directed Insertion-site Sequencing approach. This comprehensive study identified crucial genes and pathways facilitating *C. diphtheriae*’s survival at low pH. In subsequent experiments, the Ktr potassium transport system was identified as a putative key factor for maintaining pH homeostasis and growth under acidic stress. A *ktrBA* deletion strain exhibited significantly reduced growth at pH 5, which could be restored by *ktrBA* expression in *trans*. The deletion strain showed unchanged uptake and survival in macrophages compared to the wild-type, indicating that the Ktr system is not crucial for the survival of *C. diphtheriae* in phagocytes. These findings advance our understanding of *C. diphtheriae*’s pathophysiology, further delineating the intricate survival strategies of *C. diphtheriae* in hostile environments.

## Data Availability

Transposon insertion sequencing data are available at the European Nucleotide Archive (accession no. PRJEB75952, with accession no. ERR13149323 for pH 7.0 sample data and accession no. ERR13149324 for pH 5.0 sample data).

## Introduction

The genus *Corynebacterium* belongs to the phylum Actinobacteria, which comprises Gram-positive bacteria with a high G+C DNA content [1]. Within this phylum, *corynebacteria* form the CMNR group alongside the genera *Mycobacterium*, *Nocardia* and *Rhodococcus* based on a complex mycolic acid-containing cell wall structure shared by these bacteria [2]. To date, 132 taxonomically valid *Corynebacterium* species are listed at the Bacterionet website [3], including the biotechnologically important species *Corynebacterium glutamicum* and toxin-producing pathogens such as *Corynebacterium diphtheriae*, *Corynebacterium pseudotuberculosis* and *Corynebacterium ulcerans*.

*C. diphtheriae* is the aetiological agent of diphtheria. Classical diphtheria is characterized by superficial colonization of the respiratory tract epithelial layer and subsequent formation of a pseudomembrane composed of killed and decaying epithelial cells, fibrin, blood cells and bacteria. *C. diphtheriae* can also cause skin lesions and systemic infections such as bacteremia or endocarditis [4]. Furthermore, strains with arthritogenic potential have been described [5,6].

The first step of *C. diphtheriae* infection is to attach to host cells. Several adherence factors have been described, including DIP0733 [7,8] and DIP2093 [9] and SpaA-, SpaD and SpaH-type pili (for recent reviews, see Rogers *et al.* and Sangal and Burkovski [10,11]). Since iron supply is restricted in the host, *C. diphtheriae* is equipped with several iron uptake systems (Irp6A-C, DIP0582-0586 and HmuT-V, DIP1059-1062) and systems to acquire iron from haemoglobin–haptoglobin complexes (ChtC-CirA, ChtAB and HtaA-C) supporting the establishment of the bacteria within the host (for recent reviews, see Sangal and Burkovski and Sheldon and Heinrichs [11,12]).

Once in contact with the host, bacteria are an immediate target of the innate immune system. Mechanisms to survive the action of phagocytic cells described so far include secretion of toxins such as the diphtheria toxin [13] and a ribosome-binding protein with structural similarity to Shiga-like toxins [14], as well as delay of phagolysosome formation [15,16] and induction of apoptosis, pyroptosis and necrosis [14,18]. Additionally, *C. diphtheriae* exhibits considerable resistance against unfavourable conditions, such as reactive oxygen species, detergents and mechanical stress [19], which may also contribute to its pathogenesis. To improve our understanding of *C. diphtheriae* pathogenesis, studies to identify the genetic basis of its survival mechanisms during stress/host-like conditions are needed.

Recently, the first study of essential genes in *C. diphtheriae* was published, providing the most-dense Transposon Directed Insertion-site Sequencing (TraDIS) library in the phylum *Actinobacteriota* [20]. The library contains ~200,000 unique transposon mutants of *C. diphtheriae* strain ISS 3319, with an average transposon insertion density of 1 per ~12 bp, thus enabling screening of multiple mutants per gene in parallel and presenting a fantastic resource for phenotypic screening. To identify genes and proteins required for the response of *C. diphtheriae* to low pH stress, a situation relevant for survival of this human pathogen when internalized by macrophages, this library was grown in rich medium at pH 7.0 and pH 5.0.

## Methods

### Bacterial strains, plasmids and growth conditions

All strains and plasmids used in this study are listed in Table 1. *C. diphtheriae* strain ISS 3319 was cultured in Heart Infusion (HI) medium at 37 °C under shaking at 125 r.p.m. in baffled flasks. For characterization of acid stress response, the medium was adjusted to pH 5.0 using HCl. For cloning purposes, *Escherichia coli* strain DH5αMCR was grown in Lysogeny Broth (LB) medium. If appropriate, kanamycin and spectinomycin were added to the medium (50 µg ml^−1^ final concentration).

**Table 1.** Strains and plasmids used in this study

Strain designation	Description/genotype	Reference
***E. coli***		
DH5α MCR	F^-^ *endA1 supE44 thi-1* λ^-^ *recA1 gyrA96 relA1 deoR Δ(lacZYA-argF*)U169 ϕ80*ΔlacZΔ*M15 *mcrA* Δ(*mrr hsdRMS mcrBC*)	[41]
***C. diphtheriae***		
ISS 3319	Non-toxigenic clinical isolate, Biovar mitis	[5]
ISS 3319ΔktrBA	ISS 3319 carrying an unmarked *ktrBA* deletion	This study
ISS 3319 high-density transposon insertion pool		[20]
**Plasmids**		
**Designation**	**Description/genotype**	**Reference**
pCRD206	Temperature-sensitive RepA (G109D, E180K) for replication in corynebacteria, *ori_Ec_*, *sacB*, Kan^R^	[23]
pCRD206-upAL06	pCRD206 carrying upAL06 fragment downstream of *ktrA*	This study
pCRD206-upktrBupAL06	Deletion vector carrying *ktrBA* up- and downstream fragments	This study
pEKEX3	IPTG-inducible expression vector, *ptac*, *lacI*^q^, pBL1 *ori_Cg_*, ColE1 *ori_Ec_*, Spec^R^	[42]
pEKEX3-ktrBA	*ktrBA* expression plasmid	This study
pEKEX3-si	pEKEX3 carrying stabilizing insert from *Priestia* (formerly *Bacillus*) *megaterium xylR*	This study
pEKEX3-si-ktrBA	*ktrBA* expression plasmid with *xylR* silencing fragment	This study
pWH144	Delivery vector for stabilizing insert from *Priestia* (formerly *Bacillus*) *megaterium xylR*	[24]

### TraDIS: pH 5.0 transposon library screening

A previously constructed *C. diphtheriae* transposon mutant library in strain ISS 3319 comprising ~6×10^5^ mutants, representing 206,873 unique insertions [20], was grown in HI broth at pH 7.0 and pH 5.0. The media were supplemented with 25 µg ml^−1^ kanamycin and experiments were carried out in duplicate. Cells were initially grown from a starting OD_600_ of ~0.1 to mid-late exponential phase before passaging into fresh media, after which the entire culture was harvested and frozen at −80 °C for DNA extraction.

### Transposon insertion sequencing analysis

Genomic DNA extraction and transposon-gDNA junction sequencing were performed as previously described by Goodall *et al.* [20]. As we knew the density of this library, we estimated the number of mapped reads, and therefore sequencing coverage, needed to ensure sufficient sampling of the library using the equation $I=s-s({\frac{s-1}{s})}^{n}$, where *I*=insertions, *n*=number of mapped reads and *s*=the total sample size (here, 200,000). For a theoretical library of 200,000 unique insertions, assuming no loss of mutants and an equal chance of each transposon junction being sampled (sampling probability), 1 million reads are needed to ensure sampling of 99% of the library, with diminishing returns with further sequencing. Sequencing data were first processed to identify transposon tags, which, following successful identification in a two-step process allowing for up to 4 bp mismatch, were then trimmed and the remaining DNA sequence mapped to the *C. diphtheriae* ISS 3319 reference genome [21]. Sequencing data are available at the European Nucleotide Archive (accession no.: PRJEB75952) and processed data can be viewed online at our online browser: https://tradis-vault.qfab.org/. The BioTraDIS tradis_comparison.R script was used to analyse the log fold change (logFC) in read abundance per gene between conditions [22], using the default parameters with the exception of a threshold of 50 (-t 50) as the minimum number of reads per gene required for analysis.

### Mutagenesis and complementation of *ktrBA*

**Fig. 1.**
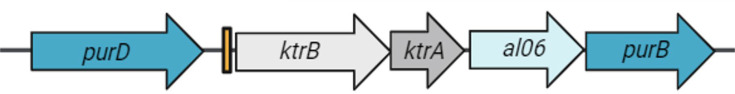
Genomic localization of the *ktrBA* locus. The genes are flanked by *purD* and *purB* with *purB* being essential under standard growth conditions. Non-coding regions were absent between *ktrB* and *purB*. A putative ribosome binding site upstream of *ktrB* is marked by an orange box.

To inactivate the Ktr potassium transport system, a deletion mutagenesis was carried out. Since the corresponding genes are located in a complex genomic situation flanked by the *pur* genes with *purB* being essential [20] and unclear promoter structures, care was taken to remove only the *ktrBA* genes without affecting other genetic structures (Fig. 1). For this purpose, a 228 bp downstream fragment was amplified by PCR using Phusion HF polymerase and the primers 5′-ATATATTCTAGAGCGTGTCTGCTATGTTTCCGAG-3′ and 5′-ATATATCCTGCAGGATCCCCAAGGTGGCAGTGTAAC-3′ leading to plasmid pCRD206-upAL06. Next, a 241 bp *ktrB* upstream fragment was PCR-amplified using the primers 5′-ATATATCACAACGTGAGAATTCCCCGCGACAACGCCTAC-3′ and 5′-TATATATCTAGAAGACTATATATTTAATTAACGAGGCGACGATACTCCG-3′ and ligated to pCRD206-upAL06 leading to pCRD206-upktrBupAL06. After sequencing to exclude unwanted mutations within the *ktrBA*-flanking fragments, mutagenesis of *C. diphtheriae* ISS 3319 was carried out as described by Okibe *et al.* [23], leading to strain ISS 3319ΔktrBA. Successful deletion was verified by PCR (data not shown).

For complementation, the *ktrBA* genes with native ribosome binding site were PCR-amplified using the primers 5′-ATATAACCTGCAGGACAATGCCCCTAGTGTTGTGAG-3′ and 5′-ATATATGAGCTCTCGGAAACATAGCAGACACG-3′ and ligated to *E. coli* shuttle vector pEKEX3 under the control of the IPTG-inducible *tac* promoter. Unfortunately, the corresponding construct proved to be unstable and prone to mutations in *E. coli* (data not shown). Therefore, a *xylR* fragment known to stabilize transcription of genes otherwise detrimental to cells [24] was amplified by PCR from plasmid pHW144 using the primers 5′-ATATATATAATGCATGATACTTTTAAATATCTAATTCAAGCTTC-3′ and 5′-ATATATATACCATTCGATGGCAAAGTTTTGAAGTGCATTTAAC-3′ and ligated to pEKEX3, leading to pEKEX3-si, which was subsequently used as the basis for *ktrBA* expression. The resulting plasmid pEKEX3-si-ktrBA was stable and sequence-verified for control (see Fig. S1, available in the online version of this article).

### Host–pathogen interaction

Human THP-1 cells were cultured in Roswell Park Memorial Institute at 1,640 medium supplemented with 10% FBS, 100 U ml^−1^ penicillin and 100 mg ml^−1^ streptomycin at 37 °C and 5% CO_2_ in a humidified cell culture incubator. Prior to infection, THP-1 cells were transferred to antibiotic-free medium with 10% FBS and differentiated into macrophage-like cells using 10 ng ml^−1^ phorbol 12-myristate 13-acetate for 24 h. Cells were infected with *C. diphtheriae* ISS 3319 and the corresponding mutant ISS 3319ΔktrBA at multiplicities of infection of 10 or left uninfected. Infection was synchronized by centrifugation at 350 r.p.m. for 5 min. After 30 min incubation, the medium was aspirated and cells were treated first with medium containing 100 µg ml^−1^ gentamicin for 1.5 h. Then, medium with a lower gentamicin concentration (10 µg ml^−1^) was added and cells were incubated until they were harvested (2, 4 or 20 h). Cells were washed three times with PBS (137 mM NaCl, 2.7 mM KCl, 10 mM Na_2_HPO_4_×12 H_2_O, 2 mM KH_2_PO_4_, pH 7.4). The supernatant was removed and cells were lysed with 500 µl distilled water. Serial dilutions of the inoculums and the lysates were plated out on Columbia agar with sheep blood (Oxoid, Wesel, Germany) to determine the number of c.f.u.

## Results

### Low pH inhibits growth of *C. diphtheriae*

**Fig. 2.**
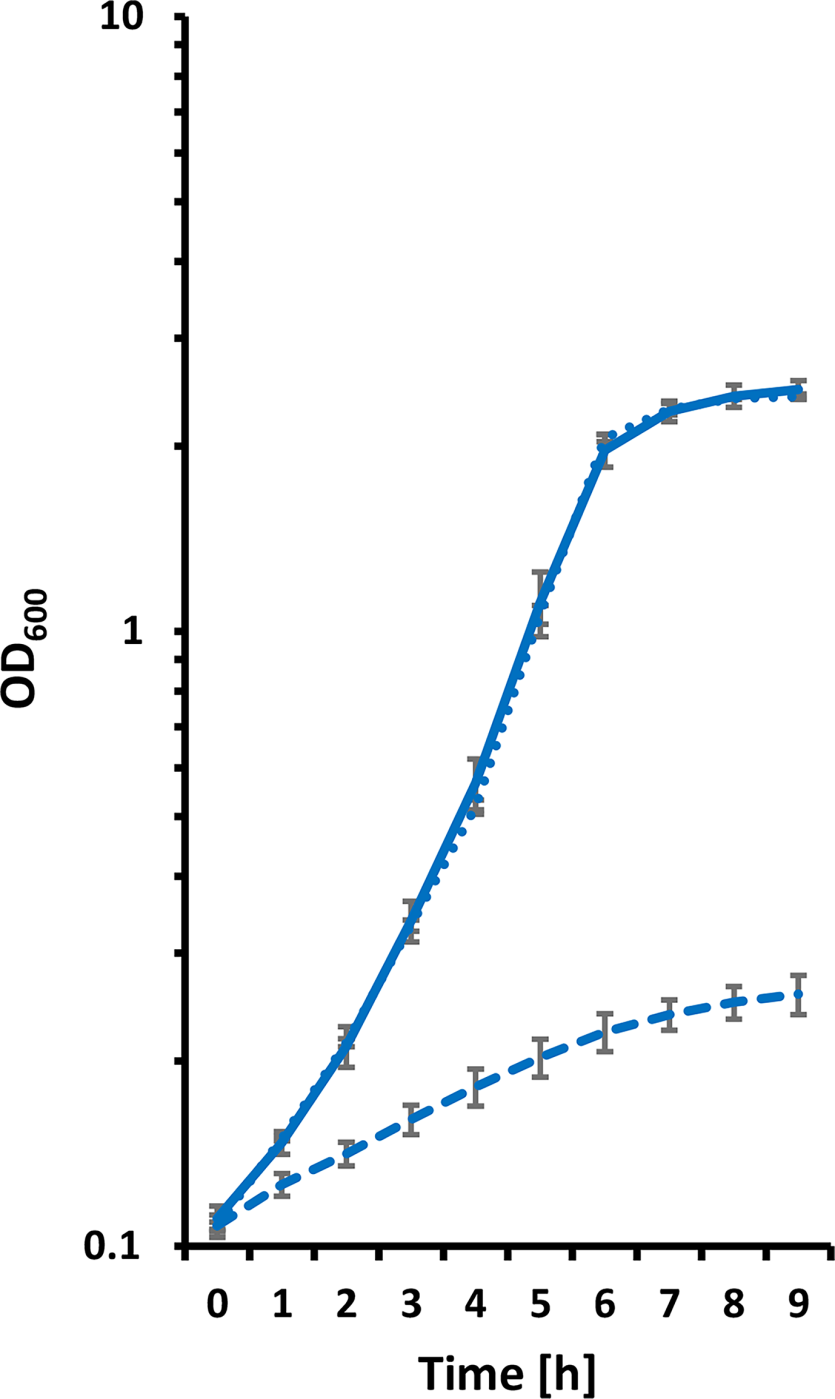
Growth of *C. diphtheriae* at different pH values. Strain ISS 3319 was grown in HI medium adjusted to pH 7.0 (solid line), 6.0 (dotted line) and 5.0 (dashed line). Error bars indicate standard deviations of triplicates.

To establish a foundation for the planned experiments, the growth of *C. diphtheriae* strain ISS 3319 was tested in rich medium adjusted to different pH levels (Fig. 2). While growth at pH 6.0 was not impaired compared with growth at pH 7.0 (103±12 min versus 101±6 min doubling time), pH 5.0 resulted in a reduced doubling time of 332±41 min. Final biomass production was influenced similarly and reached an OD_600_ of 2.48±0.12 at pH 7.0, 2.48±0.05 at pH 6.0 and 0.32±0.04 at pH 5.0. Growth was not supported at pH values below 5.0 (data not shown); therefore, subsequent experiments were carried out at pH 5.0.

### TraDIS identifies genes and proteins involved in survival at low pH

To elucidate genes essential for growth and survival at pH 5.0, a transposon mutant library containing ~200,000 unique insertions [20] was cultured in HI broth adjusted to pH 5.0 for eight generations in duplicate, or in HI broth at pH 7.0 in duplicate as a control. Samples were sequenced and processed as previously described, yielding ~2 million reads per sample (Table 2). Given the known size of this previously characterized library [20], we could estimate the required sampling depth to ensure that data absence was likely due to biological phenotypes rather than insufficient sampling. For a library of ~200,000 mutants, we estimated that 1 million reads should achieve 99% sampling coverage (see Fig. S2). In our experiments, >1.2–1.6 million reads were successfully mapped to the reference genome of each sample (Table 2), ensuring adequate coverage.

**Table 2.** Sequencing metrics per condition

Sample	No. of reads	No. of mapped reads	Unique insertions	Accession
pH 7.0	2,242,412	1,648,211	171,915	ERR13149323
pH 5.0	2,426,293	1,254,789	147,097	ERR13149324

**Fig. 3.**
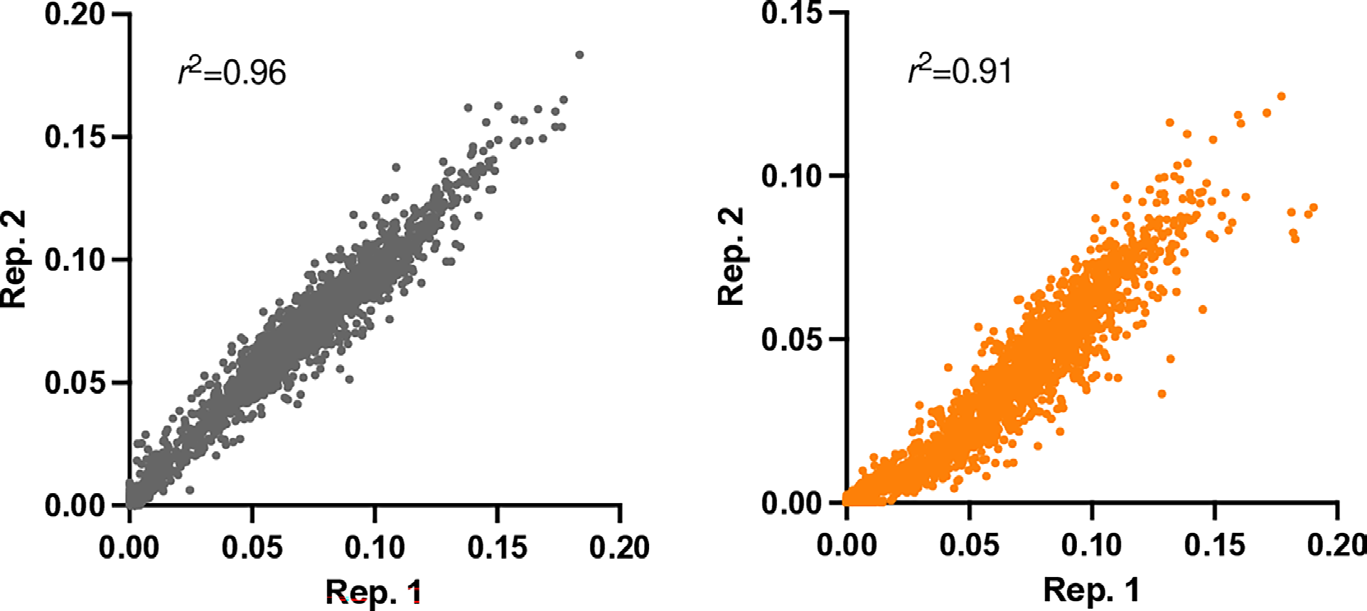
Pearson correlation analysis between replicate data. Comparison of the insertion index score per gene between replicates (grey pH 7.0, orange pH 5.0), where each point represents a different gene. The insertion index score is calculated as the number of unique insertion sites within a gene, normalized by the gene length. A higher insertion index score is demonstrative of a gene with multiple insertions, whereas a low insertion index score has few insertions (e.g. an essential gene). A high correlation between replicates indicates that equivalent insertions per gene were consistently identified between replicate data.

Comparison of the insertion index scores (the total number of insertions per gene, normalized by the gene length in bp) between replicates showed high correlation (*r*^2^=0.96, pH 7.0 *r*^2^=0.91, pH 5.0; Fig. 3), indicating strong reproducibility of the TraDIS experiment.

**Fig. 4.**
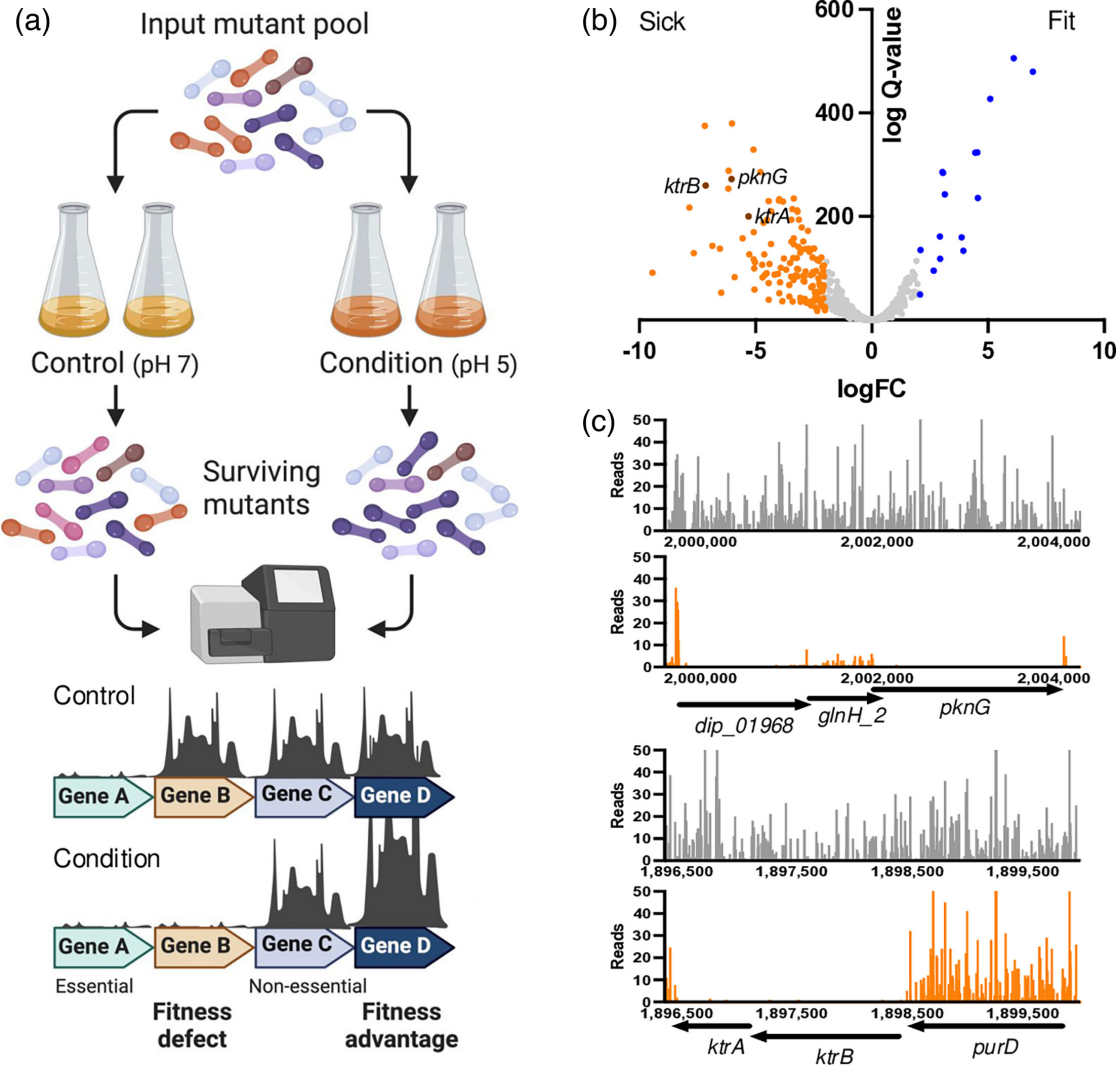
Genes required for growth at low pH. (a) Schematic overview of experimental design. (b) The logFC in read count per gene between condition replicate data. Genes with a logFC ≤ |2| and *Q*-value < 0.05 are coloured (less fit, orange; more fit, blue), while neutral loci are shown in grey. Genes represented by significantly fewer sequencing reads are indicative of mutants that are less fit and have been outcompeted during pooled growth (labelled as ‘sick’), while genes with significantly more sequencing reads are indicative of mutants that are more fit under the growth conditions and labelled as ‘fit’ accordingly. (c) Transposon insertion data of the ktrBA locus, bars correspond with the position and frequency of identified transposon insertion sites of pooled replicates following outgrowth in media (grey, pH 7.0; orange, pH 5.0).

To identify mutants with altered fitness for growth at pH 5.0, we compared the logFC in read counts per gene between pH 5.0 and control (pH 7.0) conditions. Mutants that have a fitness advantage for growth under the conditions tested are expected to proliferate and be more abundant within the overall mutant pool, which can be identified by an increase in sequencing reads/gene compared with the control sample. Conversely, mutants with a fitness defect will be outcompeted during competitive growth and will be less abundant, observed as a decrease in sequencing reads/gene compared with the control (Fig. 4a).

To identify mutants with fitness changes, we used the BioTraDIS tradis_comparison.R script, which applies the edgeR package for quantifying significant differences in read counts. Overall, we identified 135 genes that are required for growth at pH 5.0 (logFC ≤ −2, *Q*-value <0.05) and 16 genes that, when disrupted, confer a fitness advantage for growth at pH 5.0 (logFC ≥2, *Q*-value <0.05; Fig. 4b). The 15 most sick and most fit mutants (with the largest logFC between conditions) are shown in Table 3. Interestingly, 5 of the 15 transposon insertion sites with most reduced fitness are annotated to encode hypothetical proteins, highlighting potential novel factors involved in acid stress response. In addition, mutations of loci already connected to survival of acidic pH in other species were observed: (i) *pknG*, which codes for a protein kinase involved in pathogenicity in *Mycobacterium tuberculosis* [25,29] and other mycobacteria [30] and supports mycobacterial survival in acidic conditions [31], and (ii) the *ktrBA* genes, encoding a secondary active potassium importer homologous to a *Corynebacterium jeikeium* transport system, which improved pH homeostasis in acid-stressed *C. glutamicum* [44]. In addition, members of the KtrTrK transporter family have been described as key players for the homeostasis of bacterial cell physiology, osmotic resistance and bacterial fitness during host infection in several Gram-positive and Gram-negative bacteria [32].

**Table 3.** Transposon mutagenesis screen of *C. diphtheriae* ISS 3319 grown at low pH. The 15 most depleted and most enriched mutants carrying transposon insertions at pH 5.0 compared with pH 7.0 are shown (for complete results, see Table S1)

Locus tag	Gene name	Function	logFC	*Q*. value	Fitness	DIP
diphtheriae_00972	*glgE*	Alpha-1,4-glucan:maltose-1-phosphate maltosyltransferase	6.92	3.37E-145	Enriched	DIP1066
diphtheriae_00193	*deoC*_1	Deoxyribose-phosphate aldolase	6.11	5.92E-153	Enriched	DIP0273
diphtheriae_00187	*deoR*	Deoxyribonucleoside regulator	5.10	2.61E-129	Enriched	DIP0267
diphtheriae_01902	*clpC*1	ATP-dependent Clp protease ATP-binding subunit ClpC1	4.56	1.01E-71	Enriched	DIP1983
diphtheriae_01851	diptheriae_01851	Hypothetical protein	4.54	3.46E-98	Enriched	DIP1915
diphtheriae_00732	*cysQ*	3'-phosphoadenosine 5'-phosphate phosphatase	4.45	4.90E-98	Enriched	DIP0826
diphtheriae_01672	*nikA*	Nickel-binding periplasmic protein	3.94	6.11E-41	Enriched	DIP1740
diphtheriae_01675	*gsiA*	Glutathione import ATP-binding protein GsiA	3.86	7.86E-49	Enriched	DIP1743
diphtheriae_01548	diptheriae_01548	Hypothetical protein	3.13	7.91E-74	Enriched	DIP1610
diphtheriae_02236	diptheriae_02236	Universal stress protein Rv2319c	3.06	2.36E-86	Enriched	DIP2297
diphtheriae_01724	*tig*	Trigger factor	3.04	8.16E-87	Enriched	DIP1793
diphtheriae_00952	diptheriae_00952	Hypothetical protein	2.94	2.53E-36	Enriched	DIP1045
diphtheriae_00832	*coaA*	Pantothenate kinase	2.93	2.51E-49	Enriched	DIP0931
diphtheriae_00310	*senX*3	Signal-transduction histidine kinase senX3	2.67	1.89E-29	Enriched	DIP0390
diphtheriae_00228	*baeB*	Putative polyketide biosynthesis zinc-dependent hydrolase BaeB	2.09	1.84E-41	Enriched	DIP0302
diphtheriae_01868	*ktrA*	Ktr system potassium uptake protein A	−5.30	5.42E-61	Sick	DIP1930
diphtheriae_01115	*xynZ*	Endo-1,4-beta-xylanase Z	−5.56	3.78E-48	Sick	DIP1200
diphtheriae_01897	diptheriae_01897	Hypothetical protein	−5.91	1.23E-25	Sick	DIP1978
diphtheriae_01424	diptheriae_01424	Hypothetical protein	−6.01	5.45E-115	Sick	DIP1481
diphtheriae_01970	*pknG*	Serine/threonine-protein kinase PknG	−6.04	9.32E-83	Sick	DIP2053
diphtheriae_02127	diptheriae_02127	Hypothetical protein	−6.16	1.47E-87	Sick	DIP2184
diphtheriae_01935	*dacB*_2	d-alanyl-d-alanine carboxypeptidase DacB	−6.17	4.70E-77	Sick	DIP2005
diphtheriae_00659	diptheriae_00659	Hypothetical protein	−6.48	1.48E-16	Sick	DIP0729
diphtheriae_01230	diptheriae_01230	Putative gluconeogenesis factor	−6.54	3.83E-42	Sick	DIP1312
diphtheriae_00870	diptheriae_00870	Putative monoacyl phosphatidylinositol tetramannoside-binding protein LpqW	−6.86	7.70E-44	Sick	DIP0970
diphtheriae_01869	*ktrB*	Ktr system potassium uptake protein B	−7.15	6.14E-79	Sick	DIP1931
diphtheriae_02088	*lysX*	Lysylphosphatidylglycerol biosynthesis bifunctional protein LysX	−7.18	9.41E-114	Sick	DIP2138
diphtheriae_01874	*mprA*_2	Response regulator MprA	−7.67	1.16E-39	Sick	DIP1936
diphtheriae_01968	diptheriae_01968	Hypothetical protein	−7.85	3.92E-66	Sick	DIP2051
diphtheriae_01772	*clpS*	ATP-dependent Clp protease adapter protein ClpS	−9.44	2.96E-28	Sick	DIP1857

**Fig. 5.**
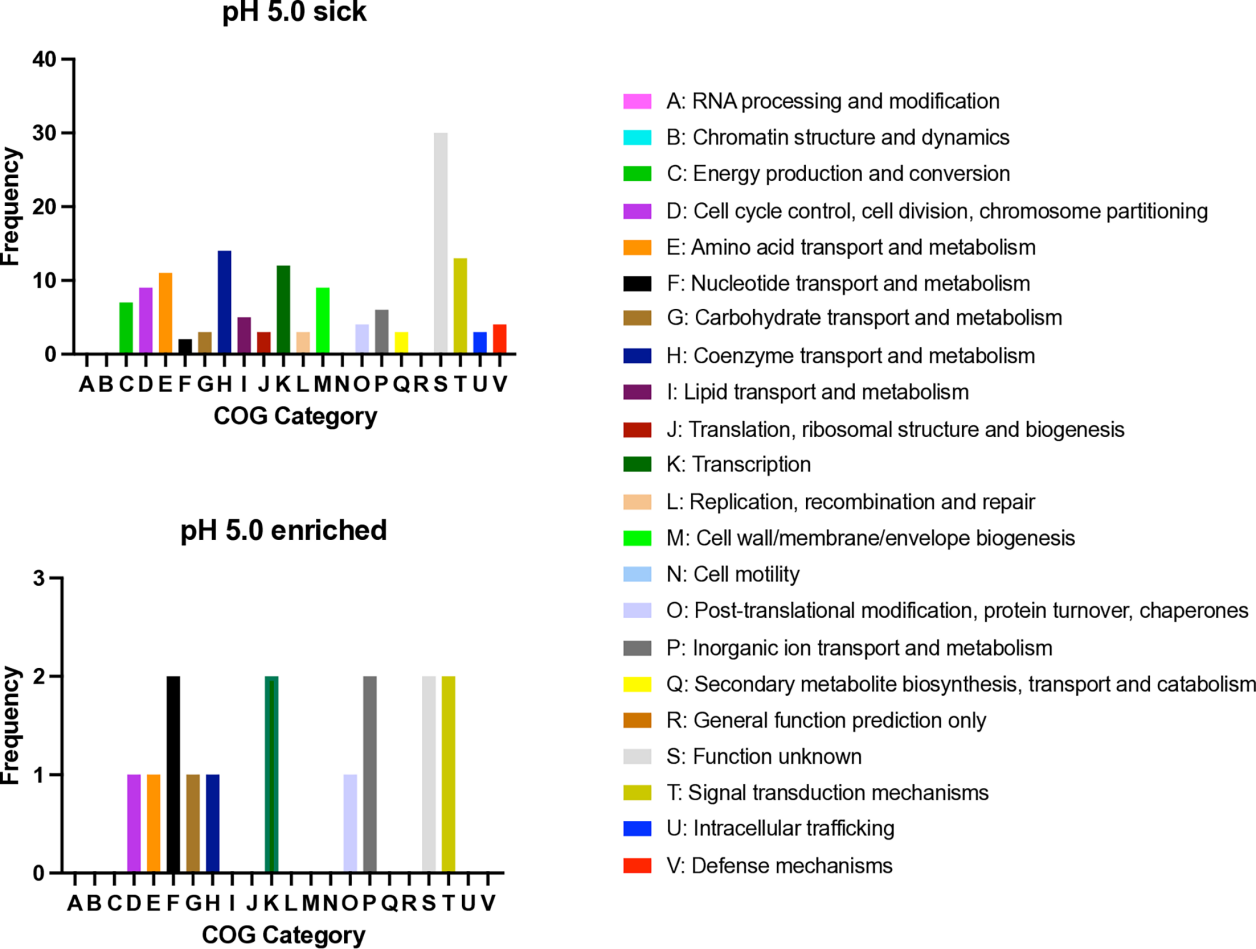
Functional classification of genes that confer a fitness advantage or disadvantage for growth at pH 5.0. Frequency of the predicted functions of the genes conferring a fitness cost or benefit when grown at pH 5.0, summarized by COG (see also Table S2).

To determine whether any overarching functions contribute to survival at pH 5.0, we reviewed the functional classification of the genes with changes in fitness. Briefly, the protein coding sequence of these genes was searched against a database of known proteins grouped by their overarching functions, termed Cluster of Orthologous Group (COG) [33] Functional classification of genes with altered fitness (required for growth or survival) at pH 5.0 revealed no predominant singular functional group. However, the largest share of genes required for survival at pH 5.0 are predicted to be of unknown function (Fig. 5).

As the transposon insertion data suggested a role for the KtrAB potassium uptake system for growth of *C. diphtheriae* at low pH (Fig. 4b, c), we investigated the role of these proteins in more detail.

### The Ktr potassium transport system is important for growth at low pH

**Fig. 6.**
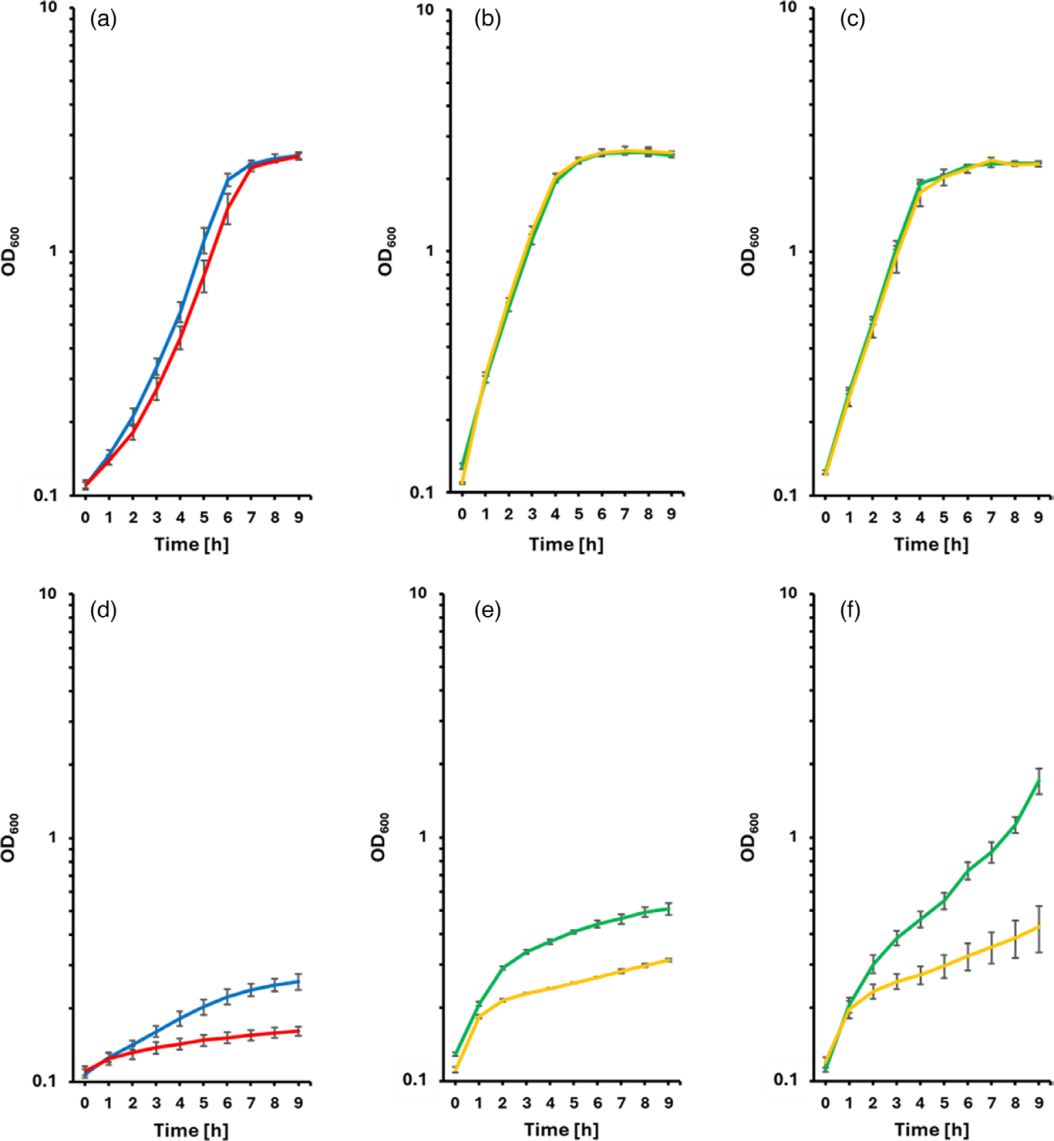
Influence of the Ktr potassium uptake system on growth of *C. diphtheriae* at acidic pH. Bacteria were grown in HI medium adjusted to pH 7 (**a-c**) and pH 5 (**d-f**), respectively. (**a, d**) blue: ISS 3319, red: ISS 3319ΔktrBA; (**b, c, e, f**) yellow: ISS 3319ΔktrBA pEKEX3-si (empty vector control), green: ISS 3319ΔktrBA pEKEX3-si-ktrBA. (**c, f**) Addition of 200 µm IPTG. Experiments were carried out in three biological replicates, each comprising three technical replicates, and standard deviations are shown as error bars.

The Ktr potassium uptake systems from *Bacillus subtilis* and *Vibrio alginolyticus* have been structurally and biochemically characterized in detail [32,34]. As these systems, *C. diphtheriae* Ktr is composed of the membrane-embedded KtrB ion channel protein and the KtrA ADP/ATP-binding regulatory protein. A deletion mutant lacking the *ktrBA* genes from start to stop codon without changing other sequences was generated and analysed in respect to its growth properties compared with the wild-type (Fig. 6). Under standard growth conditions, that is, pH 7, the deletion had almost no effect on growth (Fig. 6a): *C. diphtheriae* ISS 3319 reached a doubling time of 101±6 min, while *C. diphtheriae* ISS 3319ΔktrBA had a doubling time of 116±7 min. When strain ISS 3319ΔktrBA was transformed with pEKEX3-si or pEKEX3-si-ktrBA, improved growth was observed independent of IPTG induction (Fig. 6b, c), obviously as a vector effect. In contrast, at pH 5.0, the *ktrBA* deletion strain revealed a significantly reduced growth rate compared with the wild-type (Fig. 6d). Complementation experiments confirmed that the observed effect was due to the *ktrBA* deletion. For unknown reasons, the transformed strains showed a biphasic growth curve, and even the empty vector control showed an improved growth rate compared with the untransformed deletion strain. At pH 5.0, ISS 3319ΔktrBA pEKEX3-si reached a doubling time of 203±4 min without and 211±24 min with IPTG induction. The growth of ISS 3319ΔktrBA pEKEX3-si-ktrBA at pH 5.0 was further improved with a doubling time of 154±2 min without and 128±7 min with IPTG induction (Fig. 6e, f). Obviously, a basal expression of *ktrBA* even without IPTG induction was beneficial for growth at low pH, while overexpression due to the high copy number of plasmid-encoded genes in combination with IPTG induction led to growth exceeding that of the wild-type at pH 5.0. The positive influence of the Ktr potassium uptake system on growth at acidic conditions led to the idea that KtrAB may also influence the survival of *C. dipththeriae* in macrophages.

### Influence of the Ktr potassium transport system on survival in macrophages

*C. diphtheriae* has previously been shown to survive within the hostile environment of the phagolysosome after internalization by phagocytes [15,16]. To study the influence of KtrAB on survival in the phagolysosome, we measured the uptake and survival of ISS 3319 and ISS 3319ΔktrBA in THP-1 cells using a gentamicin protection assay. However, the internalization and survival of bacteria was not impaired, with no significant differences observed between the wild-type and *ktrBA* deletion mutant (see Fig. S2). Together, these results indicate a role for the Ktr potassium uptake system for growth at pH 5.0, but this system is not required for survival within THP-1 cells.

## Discussion

Although corynebacteria are generally highly stress-resistant [19], low pH seems to be a major growth obstacle as shown for * C. glutamicum* [35,36] and for *C. diphtheriae* as shown here. Using a TraDIS approach, we identified 135 genes in which transposon insertions caused a reduced fitness of the corresponding strains in low pH medium. Surprisingly, we also found insertions in 16 genes that enhanced fitness under these conditions. This complex response to acid stress highlights the intricate network of genes involved in bacterial adaptation to environmental challenges. Many of the corresponding proteins were involved in signal transduction, and the resulting growth advantage may be the result of complex regulatory systems as shown for mycobacterial MprAB two-component signal transduction system (TCS) that works in concert with the SenX/RegX TCS and different sigma factors, influencing and regulating phosphate starvation, general stress response and persistence in macrophages [37,40]. The involvement of multiple regulatory systems underscores the sophisticated mechanisms bacteria employ to adapt to stress conditions.

To verify the effect of transposon insertions, the *ktrBA* genes were selected for targeted mutagenesis due to their significant reduction in fitness at pH 5.0. The loss of fitness in the *C. diphtheriae ktrBA* transposon insertion strains under acidic conditions is particularly interesting in light of the fact that *C. diphtheriae* survives in macrophages via an unknown mechanism [15,16]. Notably, the corresponding deletion strain revealed a significant reduction in growth at pH 5, which was not only restored by expressing the *ktrBA* genes in trans, thereby verifying the TraDIS results, but the higher *ktrBA* copy number and *tac* promoter-driven transcription led to superior growth at pH 5 compared with the wild-type. This indicates that *C. diphtheriae* Ktr is important for pH homeostasis, similar to the corresponding *C. jeikeium* transport system [35]. The correlation between pH stress tolerance and potassium uptake systems is linked to the role of potassium in maintaining cellular homeostasis under acidic conditions. Potassium ions stabilize internal pH by counteracting acidic protons entering the cell. Potassium uptake systems, like KtrAB, ensure sufficient potassium influx to maintain optimal internal pH, membrane potential and bioenergetic processes. Without adequate potassium, cells are unable to maintain pH homeostasis, leading to impaired growth and survival under acidic stress. Therefore, efficient potassium uptake is essential for pH stress tolerance [35]. Since the *ktrBA* deletion strain exhibited unchanged uptake and survival properties in macrophages compared with the wild-type, the Ktr potassium uptake system might not be involved in *C. diphtheriae*’s survival within macrophages. This suggests that other potassium uptake systems or compensatory mechanisms might counterbalance the loss of the Ktr system. Alternatively, the macrophage environments might provide a sufficient concentration of potassium, reducing the dependency on KtrBA for survival and uptake under these conditions. Consequently, different stress responses may govern macrophage survival in *C. diphtheriae*.

The results obtained here indicate that the transposon pool-based sequencing approach provides a sensitive tool for the identification of genes important for pH stress tolerance and other environmental conditions. Compared with single strain comparisons, faster growing cells outcompete mutants with even minor growth defects in this kind of global analyses. This approach allows for a comprehensive understanding of the genetic basis of bacterial stress responses and adaptation. In conclusion, our findings highlight the complex and multifaceted nature of *C. diphtheriae*’s response to acidic stress and its interaction with host cells. The data presented here offer further avenues for investigation to understand the genetic requirement for growth and survival at low pH, as well as the species-specific adaptations that might contribute to its pathogenesis. Future studies should focus on characterizing the identified genes and their products, particularly with unknown functions, to gain deeper insights into the molecular mechanisms underlying *C. diphtheriae*’s stress response and virulence.

## Supplementary material

10.1099/mic.0.001554 Uncited Supplementary Material 1.

10.1099/mic.0.001554 Uncited Table S1.

10.1099/mic.0.001554 Uncited Table S2.

## Abbreviations

COG: Cluster of Orthologous Group
HI: heart infusion
logFC: log fold change
TCS: two component signal transduction system
TraDIS: transposon directed insertion-site sequencing
