# Supplementary material for: Genome-wide high-throughput transposon mutagenesis unveils key factors for acidic pH adaptation of Corynebacterium diphtheriae
Source: Microbiology (Reading). 2025 Apr 24;171(4):001554. doi: 10.1099/mic.0.001554 (PMC12022263; doi:10.1099/mic.0.001554)
Supplement: Uncited Supplementary Material 1. [file mic-171-01554-s001.pdf]

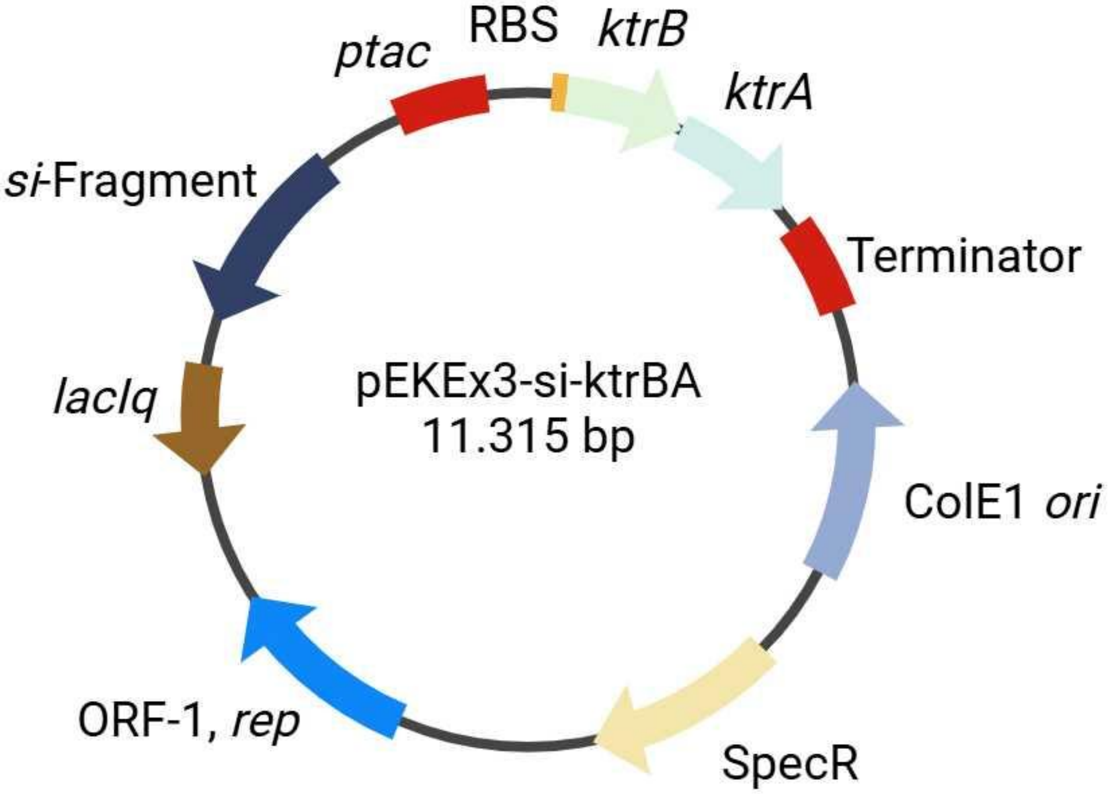

Figure S1. Vector map of *ktrBA* expression vector. The *ktrBA* genes including their native ribosome binding site (RBS) were cloned under control of the *tac* promoter (*ptac*) and the Lac repressor (encoded by *lacIq*).

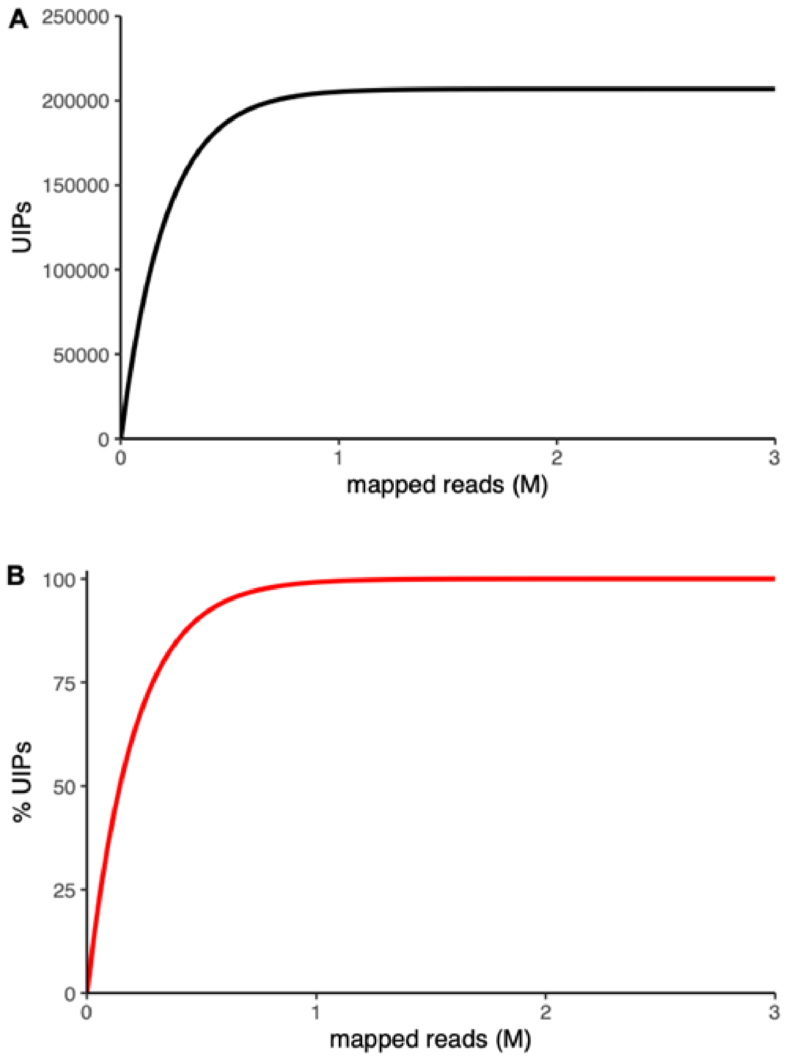

Figure S2. Estimation of the number of reads needed for full sampling of the library. Calculation of the number of mapped sequencing reads (shown in millions; M) needed to sample a given library of size *s*=200,000, presented as (A) the frequency of unique insertion points (UIPs) identified and (B) as a percentage of the total library. The equation $I = s - s\left(\frac{s-1}{1}\right)^n$ was used to estimate the number of reads needed, where *s* = 200,000, (total possible transposon insertion sites of the library); *I* = insertions identified; *n* = number of mapped reads, and assumes each transposon junction has an equal chance of being sequenced.

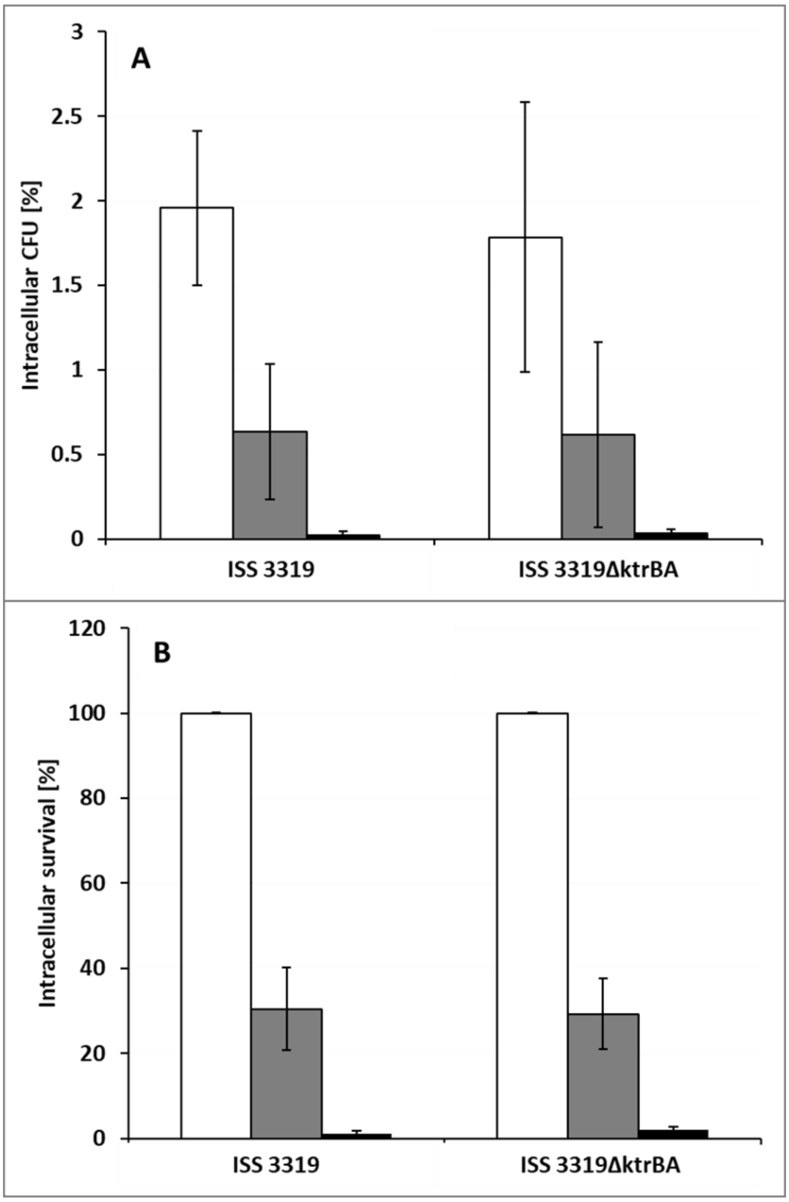

Figure S3. Quantitative analyis of viable intracellular *C. diphtheriae* in THP-1 cells. THP-1 cells were infected with wild type strain ISS 3319 and ktrBA deletion mutant ISS 3319ΔktrBA at an MOI of 10 for 30 min. To kill extracellular bacteria, cells were incubated with medium containing gentamicin for 2 (white bars), 4 (grey bars) and 20 h (black bars). Subsequently, cells were harvested, lysed and lysates were plated on blood agar plates to recover intracellular CFU. (A) Intracellular CFU as a percentage of the inoculum, (B) intracellular survival as a percentage of bacteria that were taken up after 2 h. Data shown are mean values of three independent biological replicates each performed with three technical replicates ± standard deviation.
